# Supplementary material for: Inhibitory basal ganglia nuclei differentially innervate pedunculopontine nucleus subpopulations and evoke differential motor and valence behaviors
Source: eLife. 2025 Aug 20;13:RP102308. doi: 10.7554/eLife.102308 (PMC12367300; doi:10.7554/eLife.102308)
Supplement: Supplementary file 1. [file elife-102308-supp1.pdf]

**Table 1. Statistical details of exact p-values and critical values for SNr input to ChAT+ PPN neurons**

| | | | ChAT+ | |
|---|---|---|---|---|
| **Parameter** | **Statistical Test** | **p-value** | **Rostral** | **Caudal** |
| **oIPSC Amplitude** | unpaired t-test, t=0.9041, df=33 | 0.3725 | n=15, 92.6 ± 18 pA | n=20, 115.2 ± 17 pA |
| **PPR** | unpaired t-test, t=0.1082, df=33 | 0.9145 | n=15, 0.96 ± 0.04 | n=20, 0.97 ± 0.05 |
| **Avg membrane potential** | Mann Whitney, U=87 | 0.4706 | n=11, -54.24 (-56.94 to -50.20) mV | n=19, -53.00 (-55.38 to -48.84) mV |
| **% Pre-Opto Frq** | Repeated Measures 2way ANOVA<br><br>Stimulation: F(1, 35)=81.9<br>Region: F(1, 35)=0.002052<br>Interaction: F(1, 35)=3.273 | <br><br><0.0001<br>0.9641<br>0.0790 | n=14<br><br>During: 35.62 ± 8.869 %<br>Post: 136.1 ± 7.803 % | n=23<br><br>During: 52.11 ± 8.432 %<br>Post: 119.1 ± 4.614 % |
| **ΔFrq During Opto** | Mann Whitney, U=131 | 0.3602 | n=14, -2.47 (-3.37 to -1.44) Hz | n=23, -2.10 (-3.11 to -0.84) Hz |
| **ΔRebound Frq** | Mann Whitney, U=79 | 0.0142 | n=14, 1.64 (1.00 to 2.17) Hz | n=22, 0.83 (0.42 to 1.14) Hz |

**Table 2. Statistical details of exact p-values and critical values for SNr input to Vgat+ PPN neurons**

| | | | Vgat+ | |
|---|---|---|---|---|
| **Parameter** | **Statistical Test** | **p-value** | **Rostral** | **Caudal** |
| **oIPSC Amplitude** | Mann Whitney, U=50 | 0.6005 | n=9, 76.5 (27.0 to 209.9) pA | n=13, 59.7 (37.7 to 269.7) pA |
| **PPR** | Mann Whitney, U=48 | 0.5123 | n=9, 1.01 (0.88 to 1.16) | n=13, 1.06 (0.96 to 1.15); |
| **Avg membrane potential** | unpaired t-test, t=2.468, df=13 | 0.0282 | n=5, -57.91 ± 1.630 mV | n=10, -52.00 ± 1.472 mV |
| **% Pre-Opto Frq** | Repeated Measures 2way ANOVA<br><br>Stimulation: F(1, 24)=44.63<br>Region: F(1, 24)=0.09642<br>Interaction: F(1, 24)=0.8841 | <br><br><0.0001<br>0.7588<br>0.3565 | n=7<br><br>During: 37.91 ± 15.91 %<br>Post: 100.0 ± 14.57 % | n=19<br><br>During: 35.62 ± 8.869 %<br>Post: 113.2 ± 5.187 % |
| **ΔFrq During Opto** | Mann Whitney, U=55 | 0.5336 | n=7, -5.03 (-5.55 to -1.34) Hz | n=19, -4.29 (-11.02 to -2.94) Hz |
| **ΔRebound Frq** | Mann Whitney, U=63 | >0.9999 | n=7, 0.80 (-1.31 to 2.33) Hz | n=18, 0.59 (-2.35 to 1.47) Hz |

**Table 3. Statistical details of exact p-values and critical values for SNr input to Vglut2+ PPN neurons**

| | | | Vglut2+ | |
|---|---|---|---|---|
| **Parameter** | **Statistical Test** | **p-value** | **Rostral** | **Caudal** |
| **oIPSC Amplitude** | Mann Whitney, U=29 | 0.0035 | n=13, 61.6 (19.2 to 113.9) pA | n=13, 313.5 (143.8 to 729.1) pA |
| **PPR** | Mann Whitney, U=77 | 0.7241 | n=13, 1.07 (0.86 to 1.38) | n=13, 0.92 (0.89 to 1.02) |
| **Avg membrane potential** | unpaired t-test, t=0.3838, df=25 | 0.7043 | n=11, -53.03 ± 1.815 | n=16, -52.09 ± 1.601 |
| **% Pre-Opto Frq** | Repeated Measures 2way ANOVA<br><br>Stimulation: F(1,28)=133.6<br>Region: F(1, 28)=0.5214<br>Interaction: F(1,28)=0.1765 | <br><br><br><0.0001<br>0.4762<br>0.6776 | n=13<br><br>During: 32.62 ± 10.21 %<br>Post: 100.3 ± 6.113 % | n=17<br><br>During: 24.71 ± 7.297 %<br>Post: 97.48 ± 2.228 % |
| **ΔFrq During Opto** | Mann Whitney, U=55 | 0.0343 | n=13, -3.21 (-12.21 to -1.95) Hz | n=17, -14.08 (-20.04 to -6.07) Hz |
| **ΔRebound Frq** | Mann Whitney, U=88 | 0.3627 | n=13, 0.37 (-0.027 to 0.48) Hz | n=17, -0.23 (-0.82 to 1.08) Hz |

**Table 4. Statistical details of exact p-values and critical values for GPe input to ChAT+, Vgat+, and Vglut2+ PPN neurons**

| Parameter | Statistical Test | p-value | ChAT+ | Vgat+ | Vglut2+ |
|---|---|---|---|---|---|
| **oIPSC Amplitude** | Kruskal-Wallis, H(2,37)=3.050 | 0.2176 | n=6, 22.6 (9.75 to 50.20) pA | n=19, 24.77 (6.72 to 54.17) pA | n=15, 65.71 (14.50 to 133.4) pA |
| **PPR** | Mann Whitney, U=76 | 0.0206 | | n=19, 0.97 (0.76 to 1.2) | n=15, 0.81 (0.55 to 0.93 |
| **Avg membrane potential** | Kruskal-Wallis, H(2,69)=2.963 | 0.2273 | n=25, -54.16 (-58.05 to -51.10) mV | n=18, -54.28 (-55.47 to -48.77) mV | n=29, -55.32 (-57.97 to -52.28) mV |
| **% Pre-Opto Frq** | Repeated Measures 2way ANOVA<br><br>Stimulation: F(1, 69)=25.46<br>Cell type: F(1, 69)=4.444<br>Interaction: F(1, 69)=2.805 | <br><br><0.0001<br>0.0153<br>0.0674 | n=29<br><br>During: 92.39 ± 7.59 %<br>Post: 104.10 ± 5.23 % | n=18<br><br>During: 57.70 ± 10.11 %<br>Post: 91.81 ± 6.98 % | n=24<br><br>During: 64.15 ± 7.63 %<br>Post: 104.7 ± 5.39 % |
| | Uncorrected Fisher's LSD, df=138<br><br>t=2.971<br>t=3.223<br>t=0.6177<br><br>t=0.1407<br>t=1.076<br>t=1.236 | <br><br>0.0035<br>0.0016<br>0.5378<br><br>0.8883<br>0.2838<br>0.2184 | During:<br>ChAT+ vs. Vglut2+: 28.24 (CI 9.446 to 47.03)<br>ChAT+ vs. Vgat+: 34.69 (CI 13.41 to 55.97)<br>Vglut2+ vs. Vgat+: 6.454 (CI -14.21 to 27.11)<br><br>Post:<br>ChAT+ vs. Vglut2+: -1.337 (CI -20.13 to 17.45)<br>ChAT+ vs. Vgat+: 11.58 (CI -9.702 to 32.86)<br>Vglut2+ vs. Vgat+: 12.92 (CI -7.741 to 33.58) | | |
| **ΔFrq During Opto** | Kruskal-Wallis, H(2,69)=17.21 | 0.0002 | n=25, -0.16 (-0.90 to 0.03) Hz | n=18, -1.92 (-3.85 to -0.60) Hz | n=29, -0.89 (-3.28 to -0.43) Hz |
| | Uncorrected Dunn's test | 0.0009<br>0.0002<br>0.4020 | ChAT+ vs. Vglut2+: Z=3.332<br>ChAT+ vs. Vgat+: Z=3.755<br>Vglut2+ vs. Vgat+: Z=0.8380 | | |
| **ΔRebound Frq** | Kruskal-Wallis, H(2,69)=17.21 | 0.6772 | n=8, 0.1041 (-0.52 to 0.41) Hz | n=19, 0.07082 (-0.36 to 0.57) Hz | n=7, -0.1421 (-2.58 to 0.71) Hz |
